# Development of an Antibacterial Dentin Adhesive

**DOI:** 10.3390/polym14122502

**Published:** 2022-06-19

**Authors:** Stephanie R. Lopes, Amanda G. N. Matuda, Raquel P. Campos, Ana Paula V. P. Mafetano, Ana Helena M. Barnabe, Gabriela S. Chagas, Daphne C. Barcellos, Li-Na Niu, Franklin R. Tay, Cesar R. Pucci

**Affiliations:** 1 Department of Restorative Dentistry, Institute of Science and Technology, São Paulo State University (UNESP), São José dos Campos 12245-000, São Paulo, Brazil; stephanieribeirolopes@gmail.com (S.R.L.); amanda.matuda@unesp.br (A.G.N.M.); raquel.pinto@unesp.br (R.P.C.); ana-paula.mafetano@unesp.br (A.P.V.P.M.); ana.barnabe@unesp.br (A.H.M.B.); gabriela.chagas@unesp.br (G.S.C.); 2 Department of Prosthodontics, Federal University of Espírito Santo, Vitória 29075-910, Espírito Santo, Brazil; daphnecbarcellos@hotmail.com; 3 State Key Laboratory of Military Stomatology & National Clinical Research Center for Oral Diseases & Shaanxi Key Laboratory of Stomatology, Department of Prosthodontics, School of Stomatology, The Fourth Military Medical University, Xi’an 710032, China; niulina831013@126.com; 4 Department of Endodontics, The Dental College of Georgia, Augusta University, Augusta, GA 30912, USA; tayfranklin7@gmail.com

**Keywords:** antibacterial activity, dentin, etch-and-rinse adhesive, nisin

## Abstract

Nisin is a peptide that possesses potent antibacterial properties. This study evaluated the antibacterial activity of a nisin-doped adhesive against *Streptococcus mutans*, as well as its degree of conversion and microtensile bond strength (μTBS) to dentin. Nisin was added to the adhesive Adper Single Bond 2 (3M ESPE), resulting in four groups: Control Group (Single Bond 2); Group 1% (1 wt% nisin-incorporated), Group 3% (3 wt% nisin-incorporated) and Group 5% (5 wt% nisin-incorporated). Antibacterial activity against *S. mutans* was evaluated using colony-forming unit counts (CFU). The degree of conversion was tested using FTIR. Forty human teeth were restored for μTBS evaluation. Data were statistically analyzed with ANOVA and Tukey tests at α = 0.05. The nisin-doped adhesives, for all concentrations, exhibited a significant inhibition of the growth of *S. mutans* (*p* < 0.05); Incorporation of 5% and 3% nisin decreased the degree of conversion of the adhesive (*p* < 0.05). The μTBS (in MPa): Control Group—38.3 ± 2.3^A^, Group 1%—35.6 ± 2.1^A^, Group 3%—27.1 ± 1.6^B^ and Group 5%—22.3 ± 1.0^C^. Nisin-doped adhesives exerted a bactericidal effect on S. mutans. The μTBS and degree of conversion of adhesive were not affected after incorporation of 1% nisin.

## 1. Introduction

Resin composites are the most used direct aesthetic restorative materials to date. However, the longevity of resin composite restorations is limited. These restorations fail mainly due to secondary caries [1]. The latter is caused by the infiltration and proliferation of cariogenic bacteria along the adhesive interface [2,3]. Adhesives are responsible for bonding resin composite to the dental substrate [4]. Hence, the adhesive interface is considered the Achille’s heel of the restoration [5,6]. Development of adhesives with antibacterial activities is necessary to prevent destruction of the bonded interface caused by extrinsic bacteria [1,7]. Likewise, development of adhesives with matrix metalloproteinase (MMP) inhibitory effects is highly sought after for optimizing the durability of resin-dentin bonds [8,9,10].

Adhesives with antibacterial activities may help reduce the occurrence of secondary caries [11]. Quaternary ammonium methacryloxy silane was incorporated into experimental adhesives and it was observed that the modified adhesive showed antibacterial activities without adversely affecting dentin bond strength [9]. The experimental antibacterial adhesives also demonstrated inhibitory effects on soluble MMP-9 and cathepsin K activities [9]. An antibacterial peptide, nisin, was mixed with commercial adhesives and observed antibacterial activity without compromising the bonding properties [12,13]. The antibacterial activities of the nisin-incorporated adhesives are dependent on the nisin concentration [12,13].

Nisin is an antibacterial peptide produced by *Lactococcus lactis* and is used extensively for food preservation. Nisin contains lanthionine (lantibiotic) and is effective in inhibiting the microbial growth of Gram-positive bacteria, especially those related to high food risk, such as *Staphylococcus aureus* and *S. epidermidis*, *Clostridium botulinum*, *Listeria monocytogenes* and *Streptococcus species* [14,15]. The bactericidal activity of nisin is based on depolarization of bacterial cytoplasmic membranes. Membrane depolarization results in the formation of transmembrane pores that eventually results in membrane lysis and cell death [9].

Nisin has a wide antibacterial spectrum and low animal cell cytotoxicity [15,16]. It has demonstrated potential in the prevention of dental caries [17,18] by inhibiting the growth of oral bacteria and biofilm development [19]. In addition, nisin promotes cross-links to residual cysteine. The latter, in free form, contributes to the degradation of the adhesive interface [20]. Nisin also downregulates the expression of MPP-2 and MPP-9 genes in colorectal cancer cell lines [21].

*Streptococcus mutans* is a fundamental member of the initial dental plaque biofilm and is related to the formation of dental caries [22,23]. Nisin is effective in inhibiting the proliferation of *S. mutans* [18,22]. Accordingly, the objective of the present study was to evaluate the effect of nisin incorporation on the antibacterial activity, degree of conversion and bond strength of a commercial etch-and-rinse adhesive. Three null hypotheses were tested: H01—nisin-doped adhesives do not possess antibacterial activity against *S. mutans*; H02—nisin incorporation has no effect on the degree of conversion of the adhesive; H03—nisin incorporation has no effect on the bond strength of the adhesive.

## 2. Materials and Methods

### 2.1. Adhesive Preparation

An etch-and-rinse adhesive system, Adper Single Bond 2 (Table 1), was used for nisin doping. Nisin (C143H230N42O37S7) (Handary S.A., Brussels, Belgium) was carefully weighed on a precision balance and added to the Single Bond adhesive in different concentrations. The nisin-doped adhesives and the control group were shaken using a tube agitator in the dark for 10 min at 2000 revolutions per min (rpm) [8,10], until obtaining a homogeneous and clear solution, with the particles fully incorporated, resulting in four adhesive formulations:

- Control group: control adhesive (without nisin);
- Group 1%: nisin-doped adhesive with incorporation of 1 wt% nisin;
- Group 3%: nisin-doped adhesive with incorporation of 3 wt% nisin;
- Group 5%: nisin-doped adhesive with incorporation of 5 wt% nisin.

### 2.2. Antibacterial Activities

*Streptococcus mutans*, derived from carious dentin (ATCC 25175, American Type Culture Collection, Manassas, VA, USA), was used for testing the antibacterial activities of the experimental adhesives. The *S. mutans* was cultured aerobically in Brain Heart Infusion (BHI; MilliporeSigma, St. Louis, MO, USA) broth at 37 °C. The bacteria were grown overnight, collected by centrifuge and washed three times with sterile phosphate-buffered saline (PBS). The bacteria were re-suspended in BHI and diluted to a final concentration of 1.0 × 10^7^ colony-forming units (CFU)/mL. Bacteria density was determined using a spectrophotometer (Beckman Coulter, Inc., Indianapolis, IN, USA) at an optical density of 600 nm [9,12].

Bacterial suspensions (1.0 × 10^7^ CFU/mL; 100 μL) were plated on BHI agar plates using a Drigalski spatula. Polymerized adhesive resin disks (6.5 mm diameter × 1.5 mm thick) derived from the four adhesive groups were prepared using a silicone mold and a light-emitting diode polymerization unit (Radii-cal, SDI, Victoria, Australia, 1200 mW/cm^2^) for 15 s placed individually on a bacteria-inoculated agar plate. The culture plates were incubated at 37 °C for 24 h [9,12].

For CFU counts, the disks were gently transferred to plates containing BHI agar. After the incubation period, a 150 µL aliquot of the supernatant was retrieved. The optical densities of the retrieved/samples were determined using a spectrophotometer (Spectra Count, Packard Instrument Co., Meriden, CT, USA) with a 600 nm filter. Bacterial viability was determined by counting CFUs. The process was performed three times [9,12].

### 2.3. Degree of Conversion

The degree of conversion was measured with a Fourier transform infrared spectrometer (FTIR, Spectrum 400; Perkin-Elmer, Waltham, MA, USA) with a resolution of 4 cm^−1^ in the attenuated total reflection (ATR) sampling mode. A 10 μL aliquot of the adhesive to be tested was placed on the ATR crystal. The adhesive droplet was covered with a transparent coverslip and affixed with a piece of tape to avoid evaporation of the adhesive components. The adhesives were photocured for 20 s using a light-emitting diode polymerization unit (Radii-cal, 1200 mW/cm^2^). A time-resolved spectrum collector (Spectrum TimeBase, Perkin-Elmer, MA, USA) was used for continuous and automatic collection of spectra during polymerization [10].

The spectra of a droplet of uncured adhesive and the corresponding polymerized adhesive were acquired over a spectral range of 4000 to 650 cm^−1^. The change in the band height ratios of the aliphatic carbon-carbon double bond (peak at 1638 cm^−1^) and the aromatic C=C (phenyl peak at 1608 cm^−1^) in both the uncured and cured states were monitored [10]. The degree of conversion (DC) was calculated using the formula based on the decrease in the intensity of the band ratios before and after light curing.

(1) $$\mathrm{DC}\left(\%\right)=\left(1-\left(\frac{Rcured}{Runcured}\right)\right)\times 100$$

All experiments were carried out in triplicate and the results were averaged [8,10].

### 2.4. Microtensile Bond Strength (µTBS)

Forty non-carious human molars extracted for prosthetic rehabilitation reasons were used in the study using a protocol approved by the corresponding author’s university (Local Research Ethics Committee protocol: #17729519.5.0000.0077). Ten specimens from each group were submitted to the µTBS (*n* = 10). Flat mid-coronal dentin surfaces were exposed using a cutting machine under copious water cooling (Labcut, Extec; Enfield, CT, USA), The exposed dentin surface was polished using water-cooled 600-grit abrasive water papers at 300 rpm (Politriz DP-10, Panambra, São Paulo, SP, Brazil). Surface smear layers were created using water-cooled 600-grit abrasive water papers at 300 rpm for 30 s [8,10].

Each dentin surface was etched for 15 s with a 37% phosphoric acid gel [23]. After rinsing the etchant, the excess moisture was removed with absorbent paper. Two layers of adhesives were applied on the surface with agitation for 20 s and gently air-dried for 10 s. Adhesives were light activated for 20 s (Radii-cal, 1200 mW/cm^2^) [8]. Resin composite build-ups (Filtek Z350, 3M ESPE) were placed (4 mm high, in 2 increments) and light activated for 40 s. The bonded teeth were stored in distilled water at 37 °C for 24 h [8,10].

Each bonded tooth-composite specimen was sectioned into dentin-composite resin sticks (approximately 1 mm^2^) suitable for μTBS, using a cutting machine (Labcut) at low speed and under water cooling. The six longest sticks were used for each tooth. The sticks were attached to a microtensile device in a universal testing machine (DL-200, EMIC, São José dos Pinhais, PR, Brazil). Each stick was tested in tension until failure at a crosshead speed of 0.5 mm/min using a 10 kg load cell, according to ISO 11405 Standard. The bond strength data were expressed in megapascals (MPa) [8,10].

### 2.5. Scanning Electron Microscopy Examination

The polymerized resin discs after contact with bacterial culture and nisin particles were observed using SEM to analyze the presence of *S. mutans*. The fractured surface of the dentin sticks along the bonded interface was also observed using SEM in order to investigate the morphology and to study the mode of failure after microtensile bond strength.

The specimens were placed in aluminum stubs, covered with gold/palladium (Desk II—Denton Vacuum) and were examined in a scanning electron microscope (JMS 5310—Jeol).

### 2.6. Statistical Analyses

Data obtained for the CFU counts, degree of conversion and μTBS were separately analyzed using one-way ANOVA and post-hoc Tukey tests after validating that the normality and equal variance assumptions of the corresponding data sets were not violated. Statistical significance was pre-set at α = 0.05.

## 3. Results

Table 2 shows the mean values of number of recovered bacteria (CFUs), %DC and bond strength values obtained for each group. For CFU counts of S. mutans, adhesive disks containing 1 wt%, 3 wt% and 5 wt% nisin significantly reduced viable bacteria compared with the control adhesive (*p* < 0.000). There was no significant difference in CFU counts among the three nisin-doped experimental adhesive versions (*p* > 0.05).

**Figure 1:**
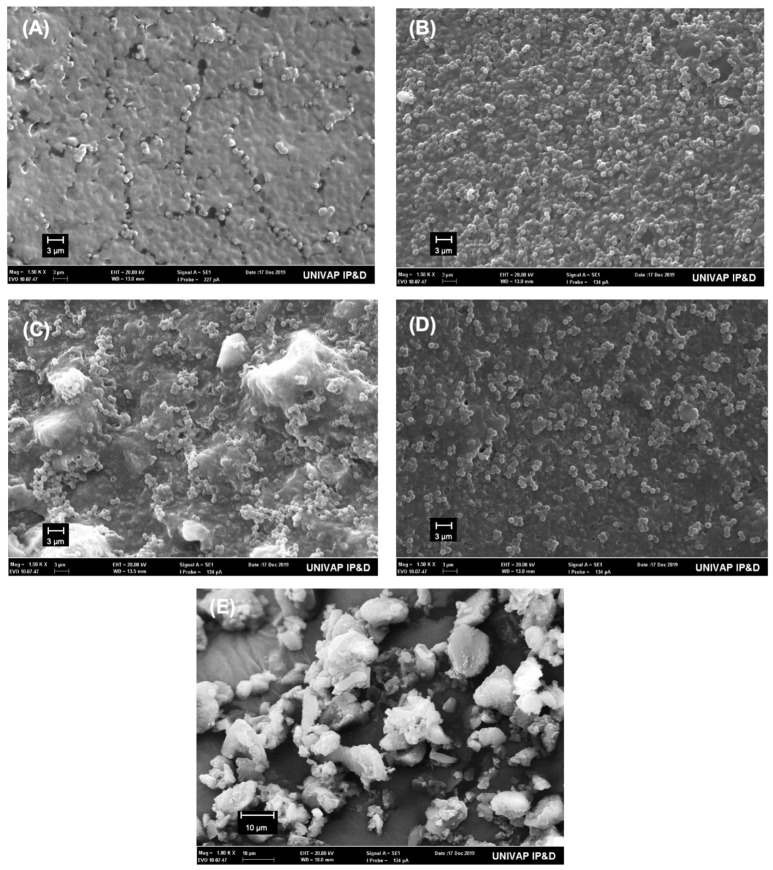
Representative SEM images of surface texture of resin polymerized disks from different groups and nisin particles. (**A**) control adhesive (without nisin); (**B**) nisin-doped adhesive with 1 wt% nisin; (**C**) nisin- doped adhesive with 3 wt% nisin; (**D**) nisin-doped adhesive with 5 wt% nisin. The CFU counts of S. mutans was roughly inversely proportional to the nisin concentration within the polymerized adhesive discs. (**E**) high magnification of the nisin particle, as we can see particle size is around 2 μm, however clusters can be larger.

Figure 1 shows typical SEM images of the surface texture of resin polymerized disks from different groups after in contact with the bacteria culture (A–D) and the nisin particles (E). The images show the presence of *S. mutans* in all groups. However, the images do not indicate whether bacteria were viable.

Incorporation of 3 wt% and 5 wt% nisin into the adhesive significantly decreased the degree of conversion when compared with the control adhesive (*p* = 0.002) and the experimental adhesive with 1 wt% nisin (*p* = 0.05).

Incorporation of 3 wt% and 5 wt% nisin into the adhesive significantly decreased the bond strength when compared with the control adhesive and 1 wt% nisin-doped adhesive. The experimental adhesive containing 5 wt% nisin showed the lowest bond strength values (*p* = 0.000).

**Figure 2:**
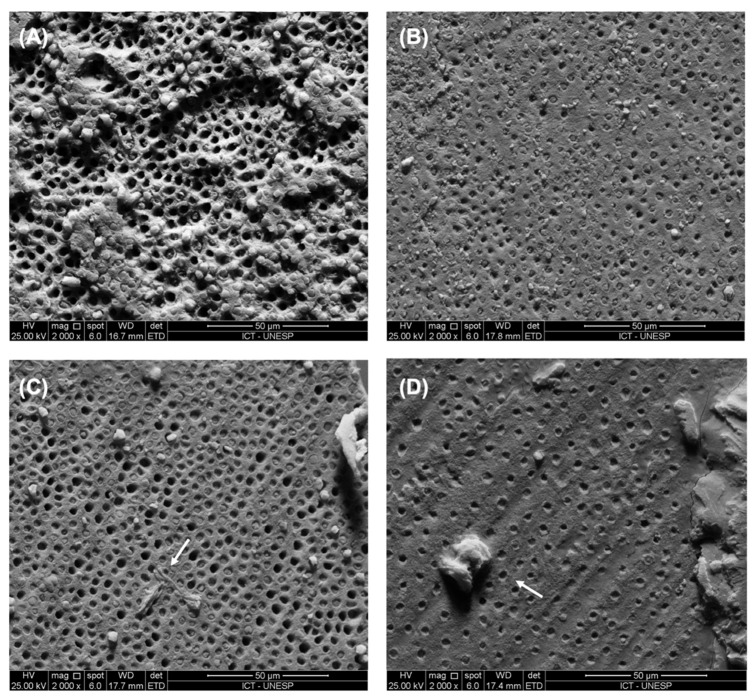
Representative failure modes observed from the dentin side of fractured composite-dentin sticks. (**A**) control group: some of the tubules were empty and had resin tags pulled out during microtensile testing. (**B**) Adhesive with 1 wt% nisin: fractured adhesive with surface of the hybrid layer showing dentin tubules. (**C**) Adhesive with 3 wt% nisin: dentin tubules with nisin clustering (arrow) between the dentin and the hybrid layer. (**D**) Adhesive with 5 wt% nisin: large nisin clustering (arrow) on the hybrid layer.

The presence of nisin clustering was identified between the dentin and the hybrid layer with the incorporation of 3 wt% and 5 wt% nisin (Figure 2C,D). The presence of these clusters made it difficult for the adhesive to infiltrate dentin, resulting in reduced bond strength. In addition, the adhesive became milky when more than 1 wt% nisin was incorporated into the adhesive.

## 4. Discussion

Placement of dental restorations creates an environmental condition that is favorable for microbial colonization along the tooth/restoration interface, which is a predisposing factor for secondary caries [1]. The surfaces of teeth with restorations are more easily colonized by microorganisms than healthy intact surfaces [24,25]. The inhibition of *S. mutans* is a crucial step in the prevention of secondary caries [18,22]. The bonded dentin interface should be properly sealed to protect the integrity of the resin composite restoration. Development of adhesives with antibacterial activity is beneficial in decreasing the risk of secondary caries and for optimizing resin-dentin bond durability. The incorporation of antibacterial peptides in dentin adhesive formulations represents one way for preventing secondary caries that is usually initiated at the tooth/resin interface.

Based on the present results, the first null hypothesis, that “nisin-doped adhesives do not possess antibacterial activity against *S. mutans*, has to be rejected. Incorporation of 1–5 wt% nisin into the etch-and-rinse adhesive significantly inhibited the growth of *S. mutans*. It is speculated that the cured nisin-doped adhesive influenced the growth, adherence and membrane integrity of *S. mutans* [12]. Even after polymerization of the nisin-doped adhesive, nisin exerts a durable and efficient antibacterial action upon direct contact with the *S. mutans* [12]. Nisin, which is a molecule that has been used as a food preservative for decades, has antimicrobial activities against cariogenic microorganisms such as *S. mutans*, *Streptocuccus sobrinus* and *Lactobacillus acidophilus* in vitro [18].

Among the different mechanisms that are responsible for the antibacterial properties of nisin, the predominant mechanism is based in its high affinity for lipid II, which is present in membranes of *S. mutans* (Gram positive bacteria). This affinity creates pores on the surface of cell membranes and interferes with cell wall biosynthesis [26,27]. The formation of pores promotes efflux of small cytoplasmic compounds, consequently causing the collapse of vital ion gradients. This ultimately results in bacterial cell death [17,18,22,26]. Nisin also promotes premature lysis of the cell wall septa of Gram-positive bacteria by displacing the autocatalytic cationic enzymes from the anionic binding sites in the bacterial cell wall [13,26]. In addition, nisin prevents the growth of bacterial spores [28] and can impair the membranes of bacteria at micromolar concentrations [29].

Nisin has potent antibacterial activity and remains stable at low pH [18,30,31]. Single Bond is a simplified etch-and-rinse adhesive system in which the primer and the bonding agent are included in a single bottle. Single Bond contains hydrophilic and acidic resin monomers, diluent monomers, photoinitiators and solvents [32]. The acidic resin monomers in its composition creates an acidic environment [12]. This may have contributed to the increase in antimicrobial activity of the nisin-doped adhesive. During bonding, the nisin-doped adhesives were actively applied on the dental substrate. This protocol was repeated twice. Agitation of the adhesive prior to light-curing could have promoted the antibacterial activity of nisin on the dentin substrate. Thus, the nisin-doped adhesive can also assume the role of a cavity disinfectant to enhance its antibacterial properties.

The CFU counts of *S. mutans* were reduced with the presence of nisin in the polymerized adhesive discs. This result is different to the findings of [12] where the activity of the adhesive-nisin discs depends on the amount of nisin, perhaps the presence of sodium chloride in nisin can did influence these results. Those authors observed, with live/dead bacteria staining, that the number of *S. mutans* decreased as the nisin concentration in the adhesive increased [12]. Unlike [12], who used nisin diluted in sodium chloride (2.5%), the present study added pure nisin to the adhesive to prevent the potential adverse effect of NaCl on bonding of the adhesive.

Because of its ability to adhere to dental substrates and direct restorative materials, *S. mutans* is the main cariogenic dental pathogen associated with the initial phase of dental caries [17]. Therefore, the inhibition of *S. mutans* can prevent the secondary caries [13]. The incorporation of nisin into a dentin adhesive can decrease the expression of genes related to extracellular polysaccharide synthesis and acidogenicity, with evidence of the loss of biofilm structure [13]. Nisin has antibacterial activity on Gram-positive pathogens [13,18]. Cariogenic bacteria generate an acidic environment by breaking down dietary carbohydrates, which creates a low pH in dental plaque. Because nisin is stable at low pH, its antibacterial activity is not compromised in the presence of cariogenic bacteria [13,18,22].

Incorporation of 3 wt% nisin into Single Bond Universal adhesive significantly decreased CFU counts of *S. mutans* [13]. However, lower nisin concentrations (1 wt% and 2 wt%) were ineffective in achieving substantial antibacterial effects on *S. mutans*. Those results were different from the present study, in which the incorporation of 1–5 wt% nisin into Single Bond 2 significantly decreased the CFU counts of *S. mutans*. Single Bond Universal is a ‘multi-mode’ adhesive that contains many ingredients in a single bottle. Apparently, nisin may have interacted with some of the components of Single Bond Universal adhesive, rendering it ineffective at 1–2 wt%. In addition, nisin with 2.5% sodium chloride (Sigma-Aldrich, St. Louis, MO, USA) was used in the study [13], on the other hand, in the present study pure nisin was used.

In the present study, the 3 wt% and 5 wt% nisin-doped adhesives had significantly lower degrees of conversion compared to the 1 wt% nisin-doped adhesive and the control adhesive. Hence, the second hypothesis, that “nisin incorporation has no effect on the degree of conversion of the adhesive,” has to be rejected. The degree of conversion is the physicochemical property that reflects the polymerization status of the resin monomers within the adhesive. This property is considered the major property of dental materials from a clinical perspective [8,10,13,33].

Nisin molecules have C=C bonds, similar to methacrylate resin monomers [13,34]. The opening of the nisin and the monomer rings may have occurred simultaneously during light-activated polymerization. Consequently, the rings may have reacted with each other during the polymerization. It is speculated that the nisin molecules may have formed a non-covalent bond with the resin monomers [13]. Incorporation of 3–5 wt% nisin into the adhesive may have reduced the crosslink densities of the polymerized adhesive. Conversely, incorporation of 1 wt% nisin into the has little effect on the degree of conversion of adhesive.

Bonding of an adhesive to the tooth tissue may be evaluated using the μTBS test [8,10,35,36]. Incorporation of 3–5 wt% nisin into the adhesive led to lower bond strength values. Hence, the third null hypothesis, that “nisin incorporation has no effect on the bond strength of the adhesive,” has to be rejected. One of the key factors of the higher bond strength of the adhesive is the degree of conversion [37,38,39]. The incorporation of 3–5 wt% nisin may have interfered with the conversion of resin monomers into polymers. This, in turn, may promote the formation of structural defects in the cross-linked polymer. On the other hand, the incorporation of 1 wt% nisin can be a good alternative for the development of bioactive adhesive systems.

Based on the antibacterial degree of conversion results and the bond strength data, it is surmised that the 1 wt% nisin-doped adhesives possess antibacterial activity without adversely affecting dentin bond strength. Future research should be conducted on the antibacterial effects of the nisin-doped adhesive against other bacteria with cariogenic potential, as well as the ability of nisin-doped adhesives to maintain dentin bond integrity over time.

## 5. Conclusions

Within the limitations of the present study, it may be concluded that the 1 wt% nisin-doped etch-and-rinse adhesive possesses antibacterial activity on *S. mutans* without compromising the degree of conversion and bond strength of the resin/dentin interface. The nisin-doped adhesive is a potential antibacterial adhesive.

## Figures and Tables

**Table 1:** Chemical composition of tested adhesive.

Adhesive	Composition	Manufacturer
Adper^TM^ Single Bond 2 Lot: 2129900256	HEMA, water, ethanol, amines, Bis-GMA, methacrylate-functional, policarboxylic acid, dimethacrylates, silanated colloidal	3M ESPE, St. Paul, MN, USA

HEMA = 2-hydroxyethyl methacrylate; Bis-GMA = bisphenol a diglycidyl dimethacrylate.

**Table 2:** Mean values and standard deviations of recovered *S. mutans* (in CFUs), % degree of conversion and μTBS values (in MPa).

Groups	CFUs (± SD) *	% Degree of Conversion (±SD) *	MPa (± SD) *
Control	0.51 × 10^7^ (±0.01) ^a^	83.5 (±3.4) ^a^	38.3 (±2.3) ^a^
1.0%	0.36 × 10^7^ (±0.03) ^b^	78.4 (±0.9) ^a^	35.6 (±2.1) ^a^
3.0%	0.34 × 10^7^ (±0.02) ^b^	52.6 (±7.2) ^b^	22.3 (±1.0) ^c^
5.0%	0.33 × 10^7^ (±0.04) ^b^	58.3 (±12.1) ^b^	27.1 (±1.6) ^b^

* ^a–c^: Same lower case letters indicate no statistical difference among groups.

## Data Availability

Data available on request.
